# *catena*-Poly[[triaqua­chlorido-μ_3_-malonato-cerium(III)] hemihydrate]

**DOI:** 10.1107/S1600536810044727

**Published:** 2010-11-06

**Authors:** Patrícia Silva, José A. Fernandes, Filipe A. Almeida Paz

**Affiliations:** a Department of Chemistry, University of Aveiro, CICECO, 3810-193 Aveiro, Portugal

## Abstract

The asymmetric unit of the title compound, {[Ce(C_3_H_2_O_4_)Cl(H_2_O)_3_]·0.5H_2_O}_*n*_, contains a Ce^3+^ atom coordinated by a chloride anion, three water mol­ecules and a malonate ligand, and one water mol­ecule of crystallization with a factor of occupancy of 50%. The malonate ligand is bonded to three different symmetry-related metal atoms yielding a one-dimensional coordination polymer running parallel to the *a* axis. A supra­molecular network composed of strong and highly directional O—H⋯O and O—H⋯Cl hydrogen bonds ensures a close and effective packing of adjacent polymeric chains.

## Related literature

For general background to coordination compounds of malonates with lanthanides, see: Cañadillas-Delgado *et al.* (2006 ▶); Doreswamy *et al.* (2003 ▶, 2005 ▶); Hernández-Molina *et al.* (2000 ▶, 2002 ▶, 2003 ▶). For previous research from our group on coordination compounds of phospho­nates, see: Cunha-Silva *et al.* (2007 ▶, 2009 ▶); Shi *et al.* (2008 ▶); Paz *et al.* (2004 ▶, 2005 ▶). For general background to the synthesis of coordination polymers using microwave heating, see: Klinowski *et al.* (2010 ▶).
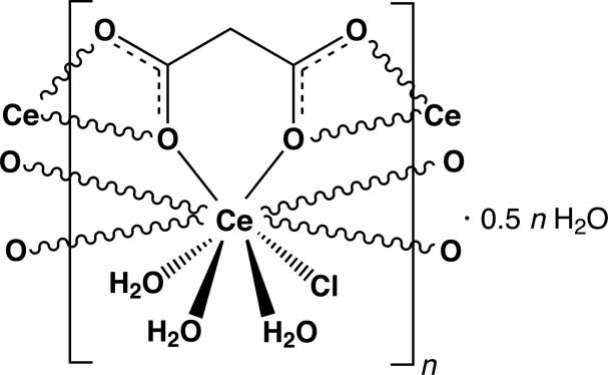


## Experimental

### 

#### Crystal data

[Ce(C_3_H_2_O_4_)Cl(H_2_O)_3_]·0.5H_2_O
*M* *_r_* = 681.34
Monoclinic, 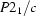

*a* = 7.6340 (2) Å
*b* = 14.3065 (3) Å
*c* = 8.7370 (2) Å
β = 99.949 (1)°
*V* = 939.87 (4) Å^3^
*Z* = 2
Mo *K*α radiation
μ = 5.13 mm^−1^
*T* = 150 K
0.26 × 0.16 × 0.16 mm

#### Data collection

Bruker X8 Kappa CCD APEXII diffractometer
Absorption correction: multi-scan (*SADABS*; Sheldrick, 1998 ▶) *T*_min_ = 0.349, *T*_max_ = 0.494
8271 measured reflections
2514 independent reflections
2481 reflections with *I* > 2σ(*I*)
*R*_int_ = 0.028

#### Refinement

*R*[*F* ^2^ > 2σ(*F* ^2^)] = 0.021
*wR*(*F* ^2^) = 0.053
*S* = 1.17
2514 reflections
143 parameters
12 restraints
H atoms treated by a mixture of independent and constrained refinement
Δρ_max_ = 1.46 e Å^−3^
Δρ_min_ = −1.77 e Å^−3^

### 

Data collection: *APEX2* (Bruker, 2006 ▶); cell refinement: *SAINT-Plus* (Bruker, 2005 ▶); data reduction: *SAINT-Plus*; program(s) used to solve structure: *SHELXTL* (Sheldrick, 2008 ▶); program(s) used to refine structure: *SHELXTL*; molecular graphics: *DIAMOND* (Brandenburg, 2009 ▶); software used to prepare material for publication: *SHELXTL*.

## Supplementary Material

Crystal structure: contains datablocks global, I. DOI: 10.1107/S1600536810044727/cv2789sup1.cif

Structure factors: contains datablocks I. DOI: 10.1107/S1600536810044727/cv2789Isup2.hkl

Additional supplementary materials:  crystallographic information; 3D view; checkCIF report

## Figures and Tables

**Table 1:** Selected bond lengths (Å)

Ce1—O1*W*	2.4580 (17)
Ce1—O3	2.4940 (16)
Ce1—O3*W*	2.5525 (18)
Ce1—O1	2.5683 (16)
Ce1—O2*W*	2.5895 (17)
Ce1—O2^i^	2.6083 (16)
Ce1—O3^ii^	2.6304 (17)
Ce1—O4^ii^	2.6487 (18)
Ce1—O1^i^	2.6793 (18)
Ce1—Cl1	2.9086 (6)

Symmetry codes: (i) 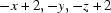; (ii) 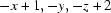.

**Table 2:** Hydrogen-bond geometry (Å, °)

*D*—H⋯*A*	*D*—H	H⋯*A*	*D*⋯*A*	*D*—H⋯*A*
O1*W*—H1*X*⋯Cl1^iii^	0.94 (1)	2.20 (2)	3.0967 (18)	159 (2)
O1*W*—H1*Y*⋯O2^iv^	0.94 (1)	1.71 (1)	2.652 (2)	174 (3)
O2*W*—H2*X*⋯Cl1^i^	0.94 (1)	2.11 (1)	3.0416 (19)	171 (3)
O2*W*—H2*Y*⋯O4*W*^v^	0.94 (1)	1.94 (2)	2.816 (4)	153 (3)
O2*W*—H2*Y*⋯O4*W*	0.94 (1)	2.00 (2)	2.793 (4)	141 (3)
O3*W*—H3*X*⋯O4^vi^	0.95 (1)	1.86 (1)	2.798 (2)	173 (3)
O3*W*—H3*Y*⋯O2*W*^ii^	0.95 (3)	1.85 (3)	2.794 (3)	173 (3)
O4*W*—H4*X*⋯Cl1^ii^	0.95 (1)	2.38 (1)	3.326 (4)	176 (6)
O4*W*—H4*Y*⋯O4^vii^	0.95 (1)	2.26 (4)	3.083 (4)	144 (5)

Symmetry codes: (i) 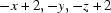; (ii) 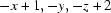; (iii) 
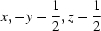
; (iv) 
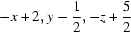
; (v) 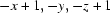; (vi) 
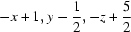
; (vii) 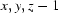.

**Fig. 1.**
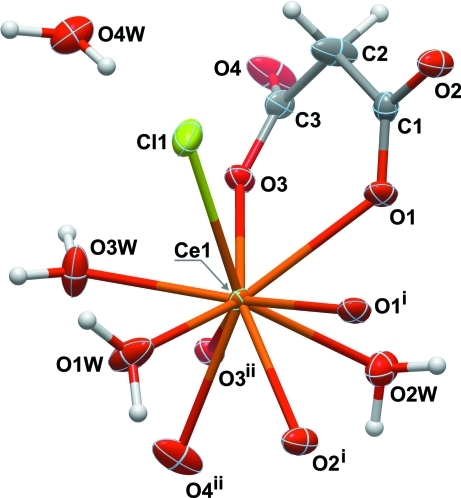
Asymmetric unit of the title compound showing all non-hydrogen atoms as displacement ellipsoids drawn at the 50% probability level and hydrogen atoms as small spheres with arbitrary radius [symmetry codes: (i)2 - x, -y, 2 - z; (ii)1 - x, -y, 2 - z]. The water molecule of crystallization, with fractional occupancy, is also depicted. For clarity, the coordination sphere of Ce1 was completed by generating by symmetry the remaining oxygen atoms.

**Fig. 2.**
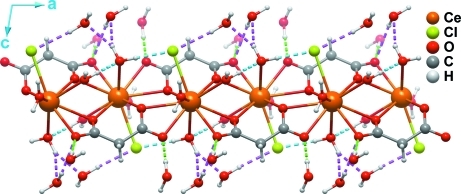
Schematic representation of the one-dimensional chain coordination polymer composing the crystal structure of the title compound. Hydrogen bonds are represented as dashed lines: blue - intra-chain interactions; pink - hydrogen bonds with the water molecule of crystallization O4W; green - inter-chain interactions establishing direct supramolecular connections between adjacent polymers.

**Fig. 3.**
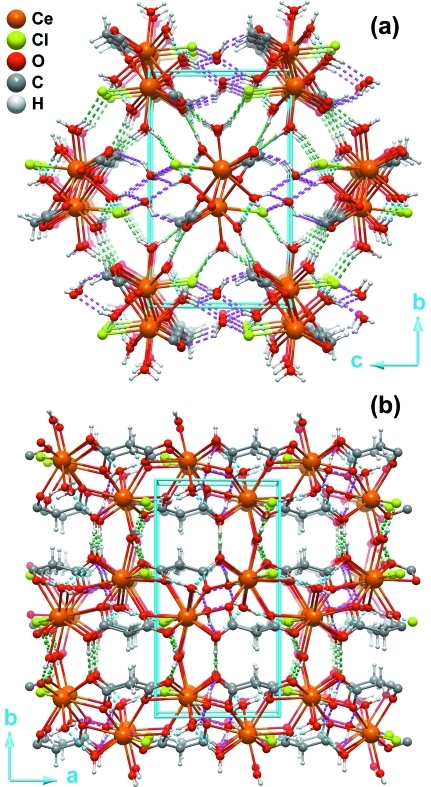
Crystal packing viewed in perspective along the (a) [100] and (b) [001] directions of the unit cell. Hydrogen bonds represented as in Figure 2.

## supplementary crystallographic information

### Comment

The hydro-ionothermal reaction between tetraethyl-*p*-xylylenebisphosphonate (texbp) and CeCl_3_^.^6H_2_O in pre-prepared homogeneous eutectic mixtures of choline chloride and malonic acid is known to lead to the phase-pure crystalline material [Ce(Hpmd)(H_2_O)] [where H_4_pmd is 1,4-phenylenebis(methylene)diphosphonic acid, the hydrolysis product of texbp] (Shi *et al.*, 2008). Following our continuous interest in the preparation and study of the properties of metal-organic frameworks based on phosphonates (Cunha-Silva *et al.*, 2009; Cunha-Silva *et al.*, 2007; Paz *et al.*, 2005; Paz *et al.*, 2004), and our recent motivation to employ microwaves as an alternative heating source (Klinowski *et al.*, 2010), we decided to test the aforementioned synthetic conditions inside a microwave reactor. The use of microwave heating instead of hydro-ionothermal conditions resulted instead in the isolation of the title compound as a by-product, being composed of a one-dimensional polymer of Ce^3+^ with malonate residues. Noteworthy, this organic ligand can be found in a number of structures comprising lanthanide centres (Cañadillas-Delgado *et al.*, 2006; Hernández-Molina *et al.*, 2000; Hernández-Molina *et al.*, 2002; Hernández -Molina *et al.*, 2003; Doreswamy *et al.*, 2003; Doreswamy *et al.* 2005) exhibiting several types of coordination modes such as unidentate, chelating and bridging.

The asymmetric unit of the title compound (see Scheme) comprises a chlorido, three water and one malonato (mal^2-^) entities coordinated to Ce^3+^, and one half-occupied water molecule of crystallization located in a generic crystallographic position: [CeCl(mal)(H_2_O)_3_]^.^0.5(H_2_O). The coordination geometry of the metallic centre can be described as a highly distorted dodecahedron (Figure 1). For example, while the Ce1—O_water_ distances range from 2.4580 (17) to 2.5895 (17) Å (see Table 1), the Ce1—Cl1 bond is considerably longer [2.9086 (6) Å]. The malonate ligand is, on the one hand, bound to the central Ce^3+^*via* distal Ce—O bonds [distances of 2.5683 (16) and 2.4940 (16) Å], leading to the formation of a six-membered chelate ring. On the other, each carboxylate establishes a physical connection with a neighbouring metallic centre *via* proximal chelations [Ce—O distances in the 2.6083 (16) to 2.6793 (18) Å range - see Table 1], thus forming the four-membered chelate rings depicted in the chemical diagram. These two different coordination modes of the malonate ligand lead to the formation of a one-dimensional coordination polymer running parallel to the [100] direction (Figure 2). The intermetallic distances within the polymer are of 4.3832 (2) and 4.5091 (2) Å.

Due to the large number of donors and acceptors, the crystal structure of the title compound is rich in strong [D···A distances in the 2.652 (2)–3.326 (4) Å range] and highly directional [〈 D—H···A larger than *ca* 141 (3)°] hydrogen bonds (see Table 2). The hydrogen bonding interactions can be divided in three different types concerning the connectivity in relation to the polymeric chain. Intra-chain hydrogen bonds add stability to the coordination polymer by connecting three adjacent metal centres through a O3W···O2W···Cl chain (blue dashed lines in Figure 2). The other two types of hydrogen bonds involve the crystal packing of adjacent coordination polymers: inter-chain interactions (green dashed lines in Figures 2 and 3) connect coordinated water molecules (O1W and O3W) of one polymer to the oxygen atoms of malonato ligands (O2 and O4, respectively) of an adjacent polymer; the second type of inter-chain interactions occur *via* the half-occupied crystallization water molecule (O4W) forming O4···O4W···Cl1 chains (pink dashed lines in Figures 2 and 3). The extensive hydrogen bonding network described above leads to a series of strong connections among adjacent coordination polymers as depicted in Figure 3.

### Experimental

The title compound was prepared following the procedure described elsewhere (Shi *et al.*, 2008), while replacing hydrothermal heating by microwave heating (Klinowski *et al.*, 2010). The reactive homogeneous suspension was transferred to a 10 ml IntelliVent reactor which was placed inside a CEM Focused Microwave^TM^ Synthesis System Discover S-Class equipment. The reaction took place with constant magnetic stirring (controlled by the microwave equipment) and by monitoring the temperature and pressure inside the vessel. Experimental conditions: i) temperature of 120 °C; ii) power of 50 W; iii) reaction time of 45 minutes of microwave irradiation. A constant flow of air (*ca* 10 psi) ensured a close control of the temperature inside the vessel. After reacting a colourless solution was obtained. The resulting solution was then left to stand at ambient temperature until large block crystals grew by slow evaporation of the solvent over a period of six months.

### Refinement

C-bound H atoms were located at their idealized positions and were included in the final structural model in riding-motion approximation with C—H distances of 0.99 Å. The isotropic displacement parameters for these atoms were fixed at 1.2×*U*_eq_ of the carbon atom to which they are attached.

All hydrogen atoms associated with the water molecules were directly located from difference Fourier maps and included in the structure with the O—H and H···H distances restrained to 0.95 (1) and 1.55 (1) Å, and with *U*_iso_ fixed at 1.5×*U*_eq_ of the O atom to which they are attached.

### Crystal data

**Table:**

[Ce(C_3_H_2_O_4_)Cl(H_2_O)_3_]·0.5H_2_O	*F*(000) = 648
*M**_r_* = 681.34	*D*_x_ = 2.408 Mg m^−^^3^
Monoclinic, *P*2_1_/*c*	Mo *K*α radiation, λ = 0.71073 Å
Hall symbol: -P 2ybc	Cell parameters from 6974 reflections
*a* = 7.6340 (2) Å	θ = 3.1–29.1°
*b* = 14.3065 (3) Å	µ = 5.13 mm^−^^1^
*c* = 8.7370 (2) Å	*T* = 150 K
β = 99.949 (1)°	Block, colourless
*V* = 939.87 (4) Å^3^	0.26 × 0.16 × 0.16 mm
*Z* = 2	

### Data collection

**Table:**

Bruker X8 Kappa CCD APEXII diffractometer	2514 independent reflections
Radiation source: fine-focus sealed tube	2481 reflections with *I* > 2σ(*I*)
graphite	*R*_int_ = 0.028
ω and φ scans	θ_max_ = 29.1°, θ_min_ = 3.7°
Absorption correction: multi-scan (*SADABS*; Sheldrick, 1998)	*h* = −7→10
*T*_min_ = 0.349, *T*_max_ = 0.494	*k* = −19→18
8271 measured reflections	*l* = −11→11

### Refinement

**Table:**

Refinement on *F*^2^	Secondary atom site location: difference Fourier map
Least-squares matrix: full	Hydrogen site location: inferred from neighbouring sites
*R*[*F*^2^ > 2σ(*F*^2^)] = 0.021	H atoms treated by a mixture of independent and constrained refinement
*wR*(*F*^2^) = 0.053	*w* = 1/[σ^2^(*F*_o_^2^) + (0.0241*P*)^2^ + 1.2954*P*] where *P* = (*F*_o_^2^ + 2*F*_c_^2^)/3
*S* = 1.17	(Δ/σ)_max_ = 0.001
2514 reflections	Δρ_max_ = 1.46 e Å^−^^3^
143 parameters	Δρ_min_ = −1.77 e Å^−^^3^
12 restraints	Extinction correction: *SHELXTL* (Sheldrick, 2008), Fc^*^=kFc[1+0.001xFc^2^λ^3^/sin(2θ)]^-1/4^
Primary atom site location: structure-invariant direct methods	Extinction coefficient: 0.0139 (5)

### Special details

**Table:**

Geometry. All e.s.d.'s (except the e.s.d. in the dihedral angle between two l.s. planes) are estimated using the full covariance matrix. The cell e.s.d.'s are taken into account individually in the estimation of e.s.d.'s in distances, angles and torsion angles; correlations between e.s.d.'s in cell parameters are only used when they are defined by crystal symmetry. An approximate (isotropic) treatment of cell e.s.d.'s is used for estimating e.s.d.'s involving l.s. planes.
Refinement. Refinement of *F*^2^ against ALL reflections. The weighted *R*-factor *wR* and goodness of fit *S* are based on *F*^2^, conventional *R*-factors *R* are based on *F*, with *F* set to zero for negative *F*^2^. The threshold expression of *F*^2^ > σ(*F*^2^) is used only for calculating *R*-factors(gt) *etc*. and is not relevant to the choice of reflections for refinement. *R*-factors based on *F*^2^ are statistically about twice as large as those based on *F*, and *R*- factors based on ALL data will be even larger.

### Fractional atomic coordinates and isotropic or equivalent isotropic displacement parameters (Å^2^)

**Table:**

	*x*	*y*	*z*	*U*_iso_*/*U*_eq_	Occ. (<1)
Ce1	0.742523 (15)	−0.079259 (8)	0.986480 (14)	0.00713 (7)	
Cl1	0.93077 (9)	−0.10017 (4)	1.30370 (7)	0.01792 (13)	
O1	0.9184 (2)	0.07240 (11)	1.0558 (2)	0.0110 (3)	
O2	1.1044 (2)	0.14538 (12)	1.23544 (19)	0.0126 (3)	
O3	0.5892 (2)	0.03721 (11)	1.12981 (19)	0.0100 (3)	
O4	0.4894 (2)	0.16117 (13)	1.2331 (2)	0.0187 (4)	
C1	0.9494 (3)	0.11752 (15)	1.1829 (3)	0.0089 (4)	
C2	0.8055 (3)	0.14023 (19)	1.2763 (3)	0.0166 (5)	
H2A	0.8375	0.1100	1.3793	0.020*	
H2B	0.8066	0.2086	1.2938	0.020*	
C3	0.6181 (3)	0.11199 (16)	1.2087 (3)	0.0113 (4)	
O1W	0.8161 (3)	−0.24629 (12)	1.0159 (2)	0.0186 (4)	
H1X	0.844 (5)	−0.2811 (19)	0.932 (2)	0.028*	
H1Y	0.844 (5)	−0.2812 (19)	1.1084 (19)	0.028*	
O2W	0.7066 (2)	0.03997 (12)	0.7602 (2)	0.0139 (3)	
H2X	0.815 (2)	0.065 (2)	0.740 (3)	0.021*	
H2Y	0.635 (3)	0.021 (2)	0.667 (2)	0.021*	
O3W	0.5195 (3)	−0.16461 (12)	1.1230 (2)	0.0185 (4)	
H3X	0.518 (5)	−0.2259 (10)	1.164 (4)	0.028*	
H3Y	0.439 (4)	−0.1266 (17)	1.167 (4)	0.028*	
O4W	0.4133 (5)	0.0545 (3)	0.5211 (4)	0.0186 (7)	0.50
H4X	0.318 (7)	0.066 (4)	0.576 (8)	0.028*	0.50
H4Y	0.435 (9)	0.109 (3)	0.465 (7)	0.028*	0.50

### Atomic displacement parameters (Å^2^)

**Table:**

	*U*^11^	*U*^22^	*U*^33^	*U*^12^	*U*^13^	*U*^23^
Ce1	0.00713 (9)	0.00740 (9)	0.00779 (9)	−0.00056 (4)	0.00394 (5)	−0.00129 (3)
Cl1	0.0202 (3)	0.0217 (3)	0.0116 (3)	−0.0073 (2)	0.0020 (2)	0.0043 (2)
O1	0.0114 (8)	0.0119 (7)	0.0108 (8)	−0.0008 (6)	0.0051 (6)	−0.0046 (6)
O2	0.0086 (8)	0.0182 (8)	0.0116 (7)	−0.0021 (6)	0.0034 (6)	−0.0050 (6)
O3	0.0094 (8)	0.0096 (7)	0.0118 (7)	−0.0012 (6)	0.0040 (6)	−0.0031 (6)
O4	0.0094 (8)	0.0192 (8)	0.0285 (10)	0.0005 (7)	0.0062 (7)	−0.0146 (7)
C1	0.0091 (10)	0.0076 (9)	0.0108 (9)	0.0003 (7)	0.0038 (8)	0.0005 (7)
C2	0.0078 (11)	0.0253 (12)	0.0180 (11)	−0.0024 (9)	0.0061 (9)	−0.0128 (10)
C3	0.0095 (10)	0.0140 (10)	0.0118 (10)	−0.0024 (8)	0.0062 (8)	−0.0034 (8)
O1W	0.0330 (11)	0.0132 (8)	0.0111 (8)	0.0097 (8)	0.0078 (7)	0.0013 (6)
O2W	0.0112 (8)	0.0178 (8)	0.0133 (8)	−0.0016 (6)	0.0036 (6)	0.0001 (6)
O3W	0.0198 (9)	0.0112 (7)	0.0288 (10)	0.0021 (7)	0.0159 (8)	0.0049 (7)
O4W	0.0189 (19)	0.0240 (18)	0.0134 (16)	0.0028 (16)	0.0043 (14)	0.0011 (14)

### Geometric parameters (Å, °)

**Table:**

Ce1—O1W	2.4580 (17)	O3—Ce1^ii^	2.6304 (17)
Ce1—O3	2.4940 (16)	O4—C3	1.256 (3)
Ce1—O3W	2.5525 (18)	O4—Ce1^ii^	2.6487 (18)
Ce1—O1	2.5683 (16)	C1—C2	1.512 (3)
Ce1—O2W	2.5895 (17)	C1—Ce1^i^	3.038 (2)
Ce1—O2^i^	2.6083 (16)	C2—C3	1.505 (3)
Ce1—O3^ii^	2.6304 (17)	C2—H2A	0.9900
Ce1—O4^ii^	2.6487 (18)	C2—H2B	0.9900
Ce1—O1^i^	2.6793 (18)	C3—Ce1^ii^	3.014 (2)
Ce1—Cl1	2.9086 (6)	O1W—H1X	0.943 (10)
Ce1—C3^ii^	3.014 (2)	O1W—H1Y	0.943 (10)
Ce1—C1^i^	3.038 (2)	O2W—H2X	0.944 (10)
O1—C1	1.271 (3)	O2W—H2Y	0.941 (10)
O1—Ce1^i^	2.6793 (18)	O3W—H3X	0.947 (10)
O2—C1	1.258 (3)	O3W—H3Y	0.95 (3)
O2—Ce1^i^	2.6083 (16)	O4W—H4X	0.947 (10)
O3—C3	1.271 (3)	O4W—H4Y	0.947 (10)
			
O1W—Ce1—O3	135.69 (6)	O3^ii^—Ce1—C3^ii^	24.86 (5)
O1W—Ce1—O3W	69.19 (6)	O4^ii^—Ce1—C3^ii^	24.56 (6)
O3—Ce1—O3W	71.11 (5)	O1^i^—Ce1—C3^ii^	137.52 (6)
O1W—Ce1—O1	134.11 (7)	Cl1—Ce1—C3^ii^	140.88 (5)
O3—Ce1—O1	65.71 (5)	O1W—Ce1—C1^i^	72.16 (6)
O3W—Ce1—O1	131.12 (6)	O3—Ce1—C1^i^	146.63 (6)
O1W—Ce1—O2W	135.43 (6)	O3W—Ce1—C1^i^	140.97 (6)
O3—Ce1—O2W	86.92 (5)	O1—Ce1—C1^i^	81.37 (6)
O3W—Ce1—O2W	132.90 (6)	O2W—Ce1—C1^i^	74.66 (6)
O1—Ce1—O2W	66.78 (6)	O2^i^—Ce1—C1^i^	24.25 (6)
O1W—Ce1—O2^i^	66.52 (6)	O3^ii^—Ce1—C1^i^	128.90 (6)
O3—Ce1—O2^i^	157.66 (5)	O4^ii^—Ce1—C1^i^	92.55 (6)
O3W—Ce1—O2^i^	126.49 (6)	O1^i^—Ce1—C1^i^	24.68 (6)
O1—Ce1—O2^i^	101.46 (5)	Cl1—Ce1—C1^i^	98.57 (4)
O2W—Ce1—O2^i^	70.93 (5)	C3^ii^—Ce1—C1^i^	113.88 (6)
O1W—Ce1—O3^ii^	116.75 (6)	C1—O1—Ce1	130.24 (15)
O3—Ce1—O3^ii^	62.43 (6)	C1—O1—Ce1^i^	93.64 (14)
O3W—Ce1—O3^ii^	67.32 (6)	Ce1—O1—Ce1^i^	118.46 (6)
O1—Ce1—O3^ii^	109.09 (5)	C1—O2—Ce1^i^	97.36 (13)
O2W—Ce1—O3^ii^	65.58 (5)	C3—O3—Ce1	141.45 (15)
O2^i^—Ce1—O3^ii^	108.72 (5)	C3—O3—Ce1^ii^	94.72 (14)
O1W—Ce1—O4^ii^	75.98 (6)	Ce1—O3—Ce1^ii^	117.57 (6)
O3—Ce1—O4^ii^	110.26 (5)	C3—O4—Ce1^ii^	94.24 (14)
O3W—Ce1—O4^ii^	73.16 (6)	O2—C1—O1	120.1 (2)
O1—Ce1—O4^ii^	143.27 (6)	O2—C1—C2	117.4 (2)
O2W—Ce1—O4^ii^	76.65 (6)	O1—C1—C2	122.5 (2)
O2^i^—Ce1—O4^ii^	68.32 (5)	O2—C1—Ce1^i^	58.39 (12)
O3^ii^—Ce1—O4^ii^	48.92 (5)	O1—C1—Ce1^i^	61.67 (12)
O1W—Ce1—O1^i^	80.87 (6)	C2—C1—Ce1^i^	175.81 (16)
O3—Ce1—O1^i^	126.38 (5)	C3—C2—C1	117.41 (19)
O3W—Ce1—O1^i^	145.13 (6)	C3—C2—H2A	107.9
O1—Ce1—O1^i^	61.54 (6)	C1—C2—H2A	107.9
O2W—Ce1—O1^i^	81.26 (6)	C3—C2—H2B	107.9
O2^i^—Ce1—O1^i^	48.93 (5)	C1—C2—H2B	107.9
O3^ii^—Ce1—O1^i^	145.80 (5)	H2A—C2—H2B	107.2
O4^ii^—Ce1—O1^i^	117.21 (5)	O4—C3—O3	119.7 (2)
O1W—Ce1—Cl1	74.57 (5)	O4—C3—C2	120.1 (2)
O3—Ce1—Cl1	77.74 (4)	O3—C3—C2	120.2 (2)
O3W—Ce1—Cl1	76.42 (5)	O4—C3—Ce1^ii^	61.20 (12)
O1—Ce1—Cl1	73.15 (4)	O3—C3—Ce1^ii^	60.43 (12)
O2W—Ce1—Cl1	139.90 (4)	C2—C3—Ce1^ii^	167.62 (17)
O2^i^—Ce1—Cl1	117.38 (4)	Ce1—O1W—H1X	120.7 (18)
O3^ii^—Ce1—Cl1	132.52 (4)	Ce1—O1W—H1Y	128.3 (18)
O4^ii^—Ce1—Cl1	143.42 (5)	H1X—O1W—H1Y	109.8 (14)
O1^i^—Ce1—Cl1	78.76 (4)	Ce1—O2W—H2X	114 (2)
O1W—Ce1—C3^ii^	94.47 (7)	Ce1—O2W—H2Y	116.0 (19)
O3—Ce1—C3^ii^	85.75 (6)	H2X—O2W—H2Y	110.2 (15)
O3W—Ce1—C3^ii^	64.67 (6)	Ce1—O3W—H3X	132.1 (19)
O1—Ce1—C3^ii^	130.83 (6)	Ce1—O3W—H3Y	116.4 (19)
O2W—Ce1—C3^ii^	72.81 (6)	H3X—O3W—H3Y	109 (3)
O2^i^—Ce1—C3^ii^	90.33 (6)	H4X—O4W—H4Y	110.0 (16)
			
O1W—Ce1—O1—C1	−84.6 (2)	C1^i^—Ce1—O3—C3	−23.8 (3)
O3—Ce1—O1—C1	46.42 (19)	O1W—Ce1—O3—Ce1^ii^	−101.07 (10)
O3W—Ce1—O1—C1	16.4 (2)	O3W—Ce1—O3—Ce1^ii^	−73.68 (8)
O2W—Ce1—O1—C1	143.9 (2)	O1—Ce1—O3—Ce1^ii^	129.82 (8)
O2^i^—Ce1—O1—C1	−152.87 (19)	O2W—Ce1—O3—Ce1^ii^	63.96 (7)
O3^ii^—Ce1—O1—C1	92.5 (2)	O2^i^—Ce1—O3—Ce1^ii^	71.47 (16)
O4^ii^—Ce1—O1—C1	138.16 (18)	O3^ii^—Ce1—O3—Ce1^ii^	0.0
O1^i^—Ce1—O1—C1	−123.6 (2)	O4^ii^—Ce1—O3—Ce1^ii^	−10.60 (9)
Cl1—Ce1—O1—C1	−37.46 (19)	O1^i^—Ce1—O3—Ce1^ii^	140.74 (6)
C3^ii^—Ce1—O1—C1	106.6 (2)	Cl1—Ce1—O3—Ce1^ii^	−153.33 (7)
C1^i^—Ce1—O1—C1	−139.21 (18)	C3^ii^—Ce1—O3—Ce1^ii^	−9.01 (7)
O1W—Ce1—O1—Ce1^i^	39.03 (11)	C1^i^—Ce1—O3—Ce1^ii^	119.68 (10)
O3—Ce1—O1—Ce1^i^	170.01 (9)	Ce1^i^—O2—C1—O1	−0.2 (2)
O3W—Ce1—O1—Ce1^i^	139.96 (7)	Ce1^i^—O2—C1—C2	179.99 (18)
O2W—Ce1—O1—Ce1^i^	−92.54 (8)	Ce1—O1—C1—O2	133.00 (19)
O2^i^—Ce1—O1—Ce1^i^	−29.27 (8)	Ce1^i^—O1—C1—O2	0.2 (2)
O3^ii^—Ce1—O1—Ce1^i^	−143.90 (7)	Ce1—O1—C1—C2	−47.2 (3)
O4^ii^—Ce1—O1—Ce1^i^	−98.25 (10)	Ce1^i^—O1—C1—C2	180.0 (2)
O1^i^—Ce1—O1—Ce1^i^	0.0	Ce1—O1—C1—Ce1^i^	132.79 (18)
Cl1—Ce1—O1—Ce1^i^	86.13 (7)	O2—C1—C2—C3	176.4 (2)
C3^ii^—Ce1—O1—Ce1^i^	−129.82 (8)	O1—C1—C2—C3	−3.4 (4)
C1^i^—Ce1—O1—Ce1^i^	−15.61 (7)	Ce1^ii^—O4—C3—O3	−15.7 (2)
O1W—Ce1—O3—C3	115.4 (2)	Ce1^ii^—O4—C3—C2	165.9 (2)
O3W—Ce1—O3—C3	142.8 (3)	Ce1—O3—C3—O4	163.95 (17)
O1—Ce1—O3—C3	−13.7 (2)	Ce1^ii^—O3—C3—O4	15.9 (2)
O2W—Ce1—O3—C3	−79.6 (2)	Ce1—O3—C3—C2	−17.7 (4)
O2^i^—Ce1—O3—C3	−72.1 (3)	Ce1^ii^—O3—C3—C2	−165.7 (2)
O3^ii^—Ce1—O3—C3	−143.5 (3)	Ce1—O3—C3—Ce1^ii^	148.1 (2)
O4^ii^—Ce1—O3—C3	−154.1 (2)	C1—C2—C3—O4	−146.9 (2)
O1^i^—Ce1—O3—C3	−2.8 (3)	C1—C2—C3—O3	34.7 (3)
Cl1—Ce1—O3—C3	63.1 (2)	C1—C2—C3—Ce1^ii^	−54.5 (8)
C3^ii^—Ce1—O3—C3	−152.5 (2)		

Symmetry codes: (i) −*x*+2, −*y*, −*z*+2; (ii) −*x*+1, −*y*, −*z*+2.

### Hydrogen-bond geometry (Å, °)

**Table:**

*D*—H···*A*	*D*—H	H···*A*	*D*···*A*	*D*—H···*A*
O1W—H1X···Cl1^iii^	0.94 (1)	2.20 (2)	3.0967 (18)	159 (2)
O1W—H1Y···O2^iv^	0.94 (1)	1.71 (1)	2.652 (2)	174 (3)
O2W—H2X···Cl1^i^	0.94 (1)	2.11 (1)	3.0416 (19)	171 (3)
O2W—H2Y···O4W^v^	0.94 (1)	1.94 (2)	2.816 (4)	153 (3)
O2W—H2Y···O4W	0.94 (1)	2.00 (2)	2.793 (4)	141 (3)
O3W—H3X···O4^vi^	0.95 (1)	1.86 (1)	2.798 (2)	173 (3)
O3W—H3Y···O2W^ii^	0.95 (3)	1.85 (3)	2.794 (3)	173 (3)
O4W—H4X···Cl1^ii^	0.95 (1)	2.38 (1)	3.326 (4)	176 (6)
O4W—H4Y···O4^vii^	0.95 (1)	2.26 (4)	3.083 (4)	144 (5)

Symmetry codes: (iii) *x*, −*y*−1/2, *z*−1/2; (iv) −*x*+2, *y*−1/2, −*z*+5/2; (i) −*x*+2, −*y*, −*z*+2; (v) −*x*+1, −*y*, −*z*+1; (vi) −*x*+1, *y*−1/2, −*z*+5/2; (ii) −*x*+1, −*y*, −*z*+2; (vii) *x*, *y*, *z*−1.
